# Supplementary material for: DNA methylation signatures follow preformed chromatin compartments in cardiac myocytes
Source: Nat Commun. 2017 Nov 21;8:1667. doi: 10.1038/s41467-017-01724-9 (PMC5698409; doi:10.1038/s41467-017-01724-9)
Supplement: Supplementary file 1 — Supplementary Information [file 41467_2017_1724_MOESM1_ESM.pdf]

Supplementary Figures

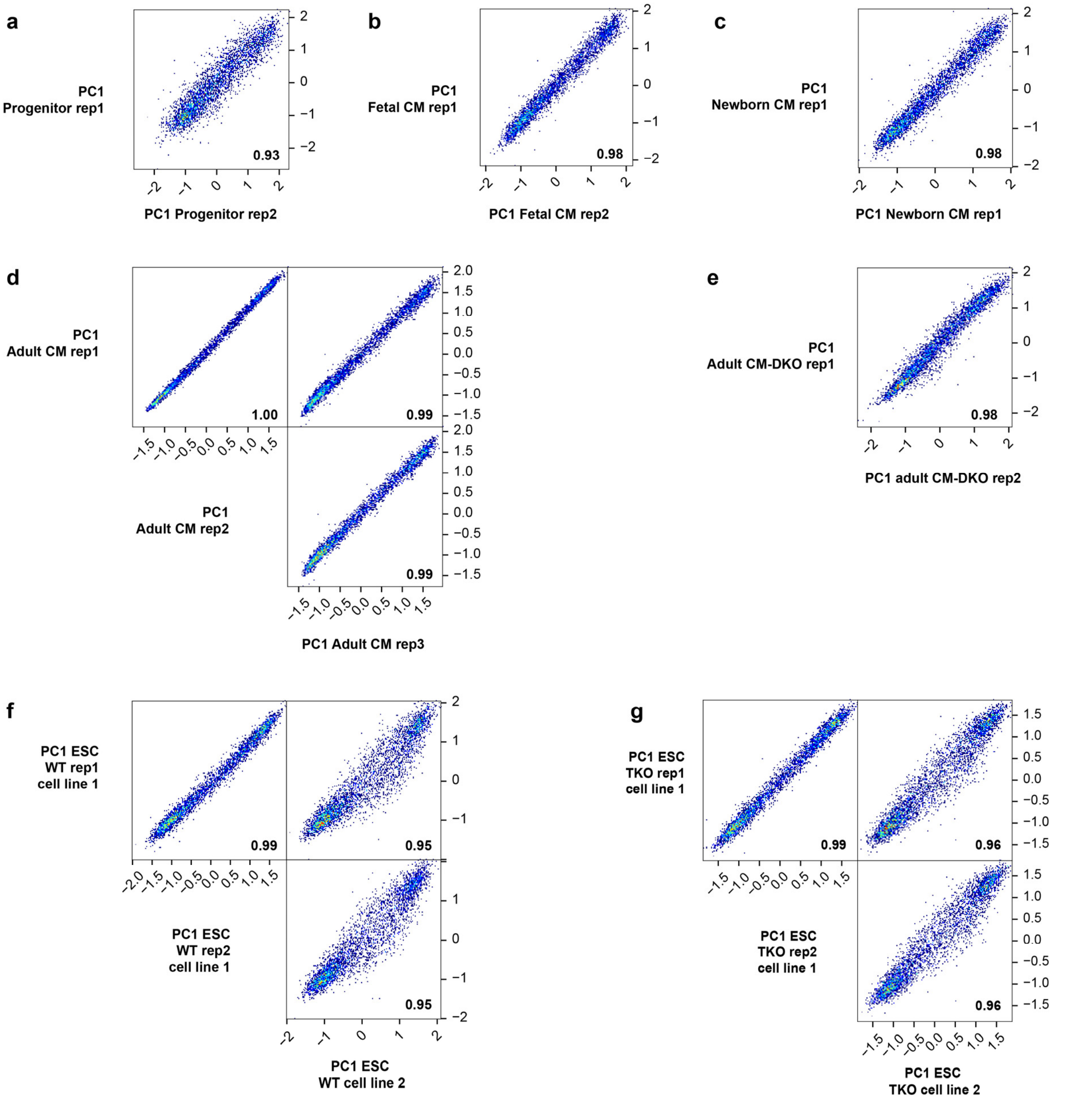

Supplementary Figure 1 │ Correlation of PC1 values of biological replicates prior of merging

Data shown are scatter plots of PC1 values of biological replicates. **a-e**, Depicted are results of different developmental stages of cardiac myocytes (**a**, cardiac progenitor; **b**, fetal, E14; **c**, newborn, P1; **d**, adult) and of cardiac myocytes after ablation of DNMT3A/B (**e**, CM-DKO, *Dnmt3a*$^{-/-}$/*Dnmt3b*$^{-/-}$). **f, g,** Scatter plots of wild type ES cells (**f**) and ES cells with ablation of DNMT1 and DNMT3A/B (**g**, ESC-TKO, *Dnmt1*$^{-/-}$/*Dnmt3a*$^{-/-}$/*Dnmt3b*$^{-/-}$) are shown for two independent cell lines (cell line 1 and 2). Numbers represent Pearson correlation coefficients of biological replicates.

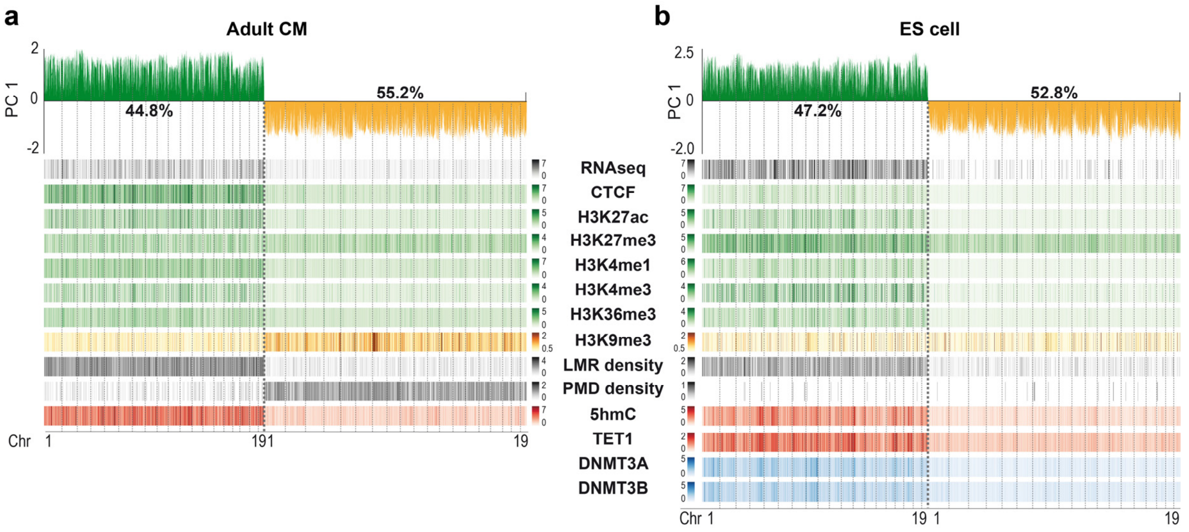

Supplementary Figure 2 │ Interplay of chromatin organization, chromatin state and DNA methylation in mouse adult CM and ES cell

a,b, Heat maps of gene expression (RNA-seq, FPKM), CTCF (RPKM), histone modifications (RPKM), DNA modifying enzymes (TET1, DNMT3A and B) and 5-hydroxymethylcytosine (5hmC) were depicted together with the density of LMRs and PMDs per 40kb bin. Bins of autosomes separated by A/B-compartment status and sorted according to chromosomal location (A, PC1 >0; B; PC1 <0). PC1 values were shown in the upper parts of the figure. Percentages indicate the distribution of A/B-compartments in mouse adult CM **(a)** and ES cells **(b)**. Data shown are merged from n=1-2 replicates (see Supplementary Table 1 and 2). Abbreviations: RPKM, Reads Per Kilobase per Million; FPKM, Fragments Per Kilobase per Million

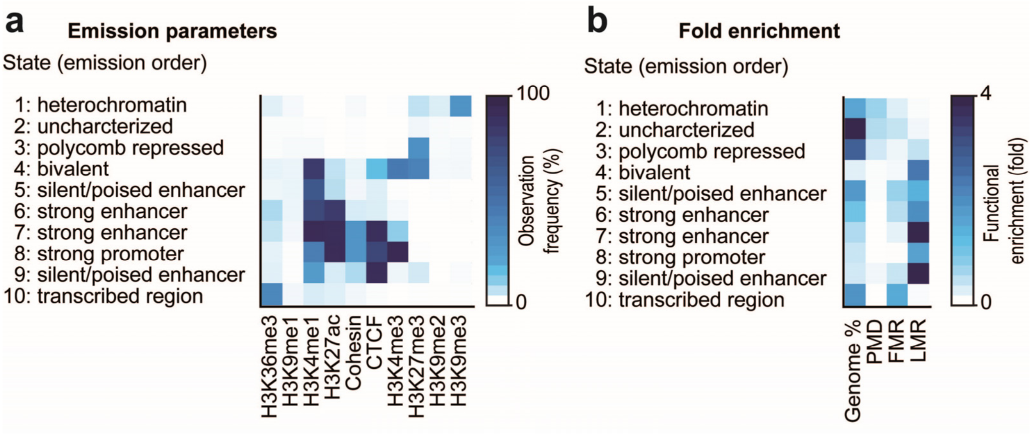

Supplementary Figure 3 | Annotation of chromatin states using automated chromatin segmentation in adult cardiac myocytes.

(a,b) Shown are heat maps for model parameters derived by ChromHMM. **a,** Chromatin states were learned using a multi variant hidden Markov model on the basis of genome wide occurrence of combinations of different histone marks, CTCF and cohesin. States with frequent observation of H3K9me3 represent heterochromatin, with H3K27me3 polycomb repressed regions, with H3K4me3 and H3K27me3 bivalent regions, with H3K4me1 silent/poised enhancer, with H3K27ac and H3K4me1 strong enhancer, with H3K4me3 promoter and with H3K36me3 actively transcribed regions. Enhancers were further separated according to presence or absence of CTCF. **b,** The enrichment of chromatin states in partially methylated domains (PMDs), fully methylated regions (FMRs) and low methylated regions (LMRs) indicates that PMDs span mainly inactive chromatin, FMRs transcribed regions and LMRs enhancers.

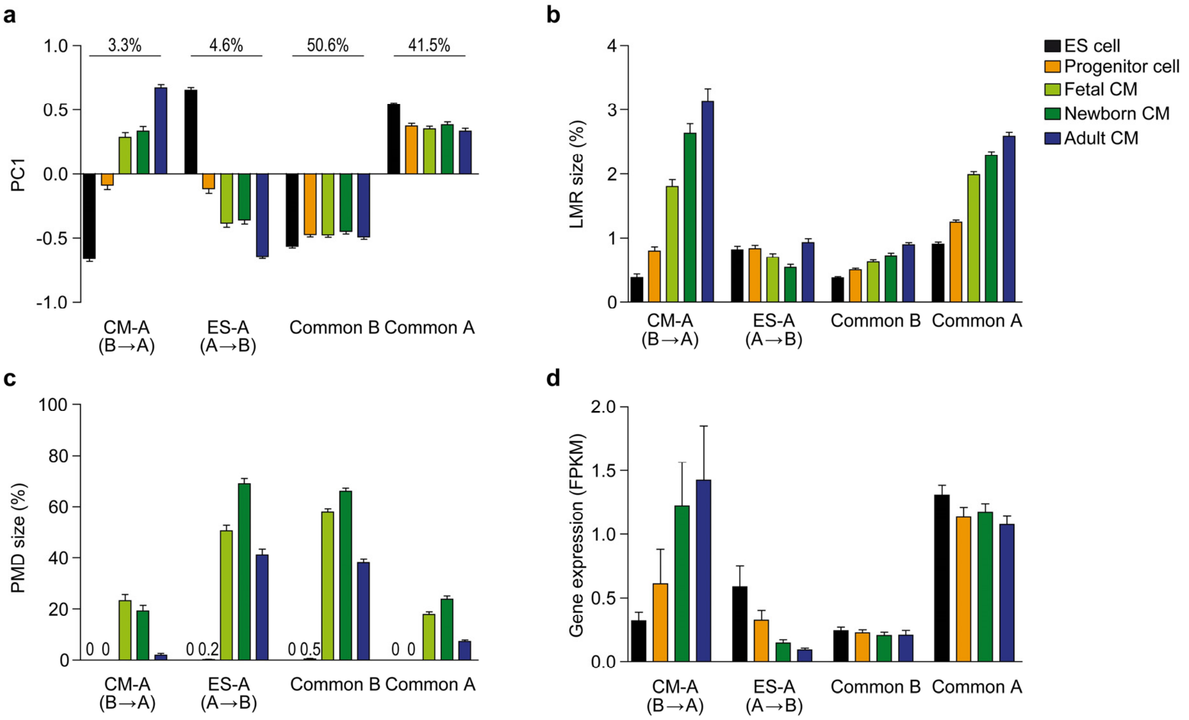

Supplementary Figure 4 │ Global dynamics of chromatin architecture, CpG-methylation and transcription during differentiation and maturation of CM

a, Depicted are dynamic and stable A/B-compartments during differentiation and maturation of cardiac myocytes (A, PC1 > 0; B, PC1 < 0). Numbers indicate genome wide portions. **b,c,d,** Cumulative size of low methylated regions (LMRs) and partially methylated domains (PMDs) as well as gene expression (FPKM) in dynamic and stable A- and B-compartments. Data shown are from n=1-3 replicates (see Supplementary Table 1 and 2). Shown are mean ± SEM. Abbreviations: FPKM, Fragments Per Kilobase per Million

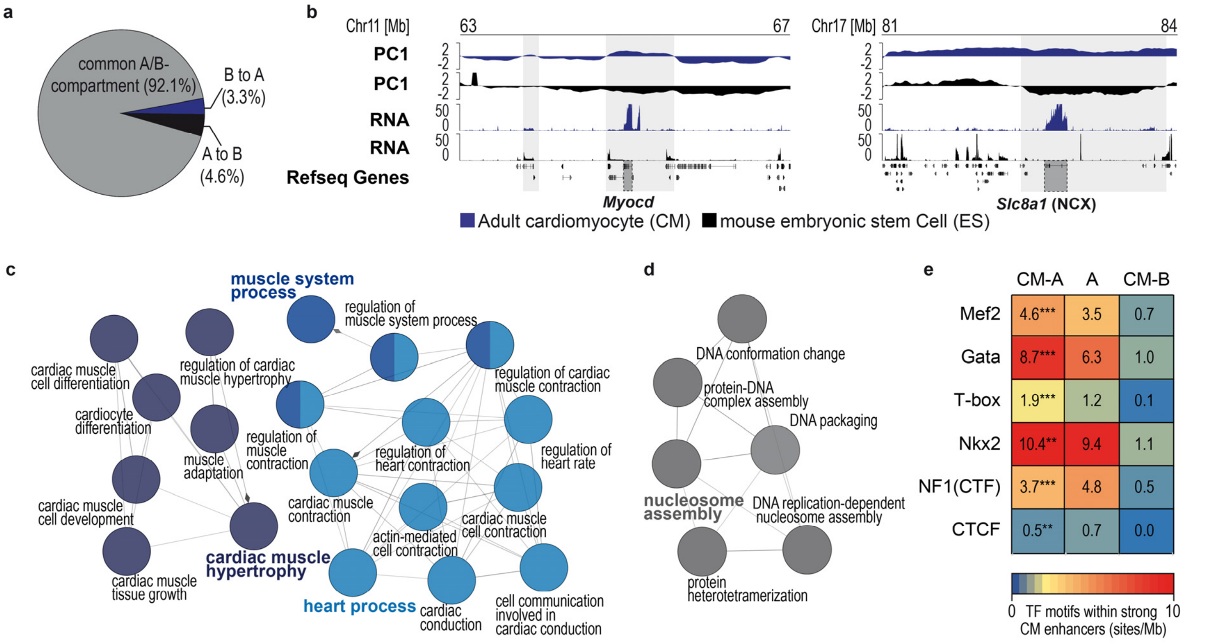

Supplementary Figure 5 │ Cell type specificity of chromatin compartments

a, Pie chart of compartments (40kb bins) exhibiting identical or differential A/B-status in CM (CM-A) versus ES cells (ES-A). Blue represents compartments with A status in CM but not in ES cells and black A status in ESC and not in CM. **b,** Representative regions with differential A/B-status (A, PC1 > 0; B, PC1 <0) in CM versus ES cells (grey background). Abbreviations: *Myocd*, Myocardin; *Slc8a1* (NCX1), Sodium-Calcium-Exchanger. Shown are PC1 values and RNA expression (FPKM) **c,d,** Gene ontology analysis of genes situated in CM-A **(c)** or in ES-A **(d)**. Shown are terms with P values < 10^{-6} (Bonferroni corrected hypergeometric test). **e,** Frequency of transcription factor motifs within strong enhancers positive for H3K27ac and H3K4me1 of CM-A, Common-A and CM-B. ** P < 0.01 and *** P < 0.001 as compared to Common-A (A), Chi-square. Abbreviations: FPKM, Fragments Per Kilobase per Million. Data shown are merged from n=1-3 replicates (see Supplementary Table 1 and 2).

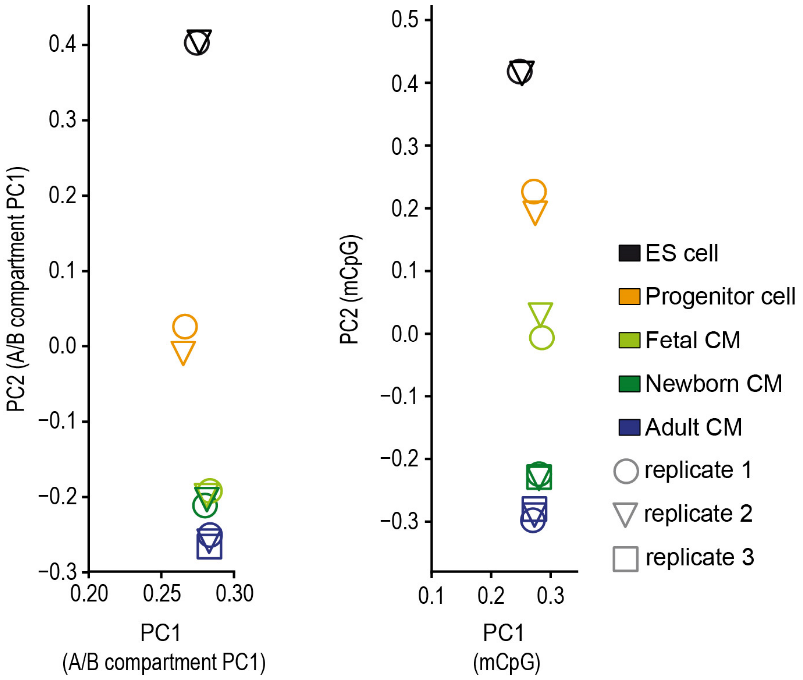

Supplementary Figure 6 │ Principal component analysis of A/B-compartment values and DNA methylation of independent replicates (Replication of Figure 2d)

Genome-wide principle component analysis (PCA) of A-/B-compartment values results in a tight cluster of differentiated cardiac myocytes and distant pluripotent ES and multipotent progenitor cells (left graph). Performing PCA analysis of base-pair resolution CpG methylation data results in a trajectory of CM differentiation and maturation with the smallest distance between postnatal stages (right graph). Data shown are results from independent biological replicates.

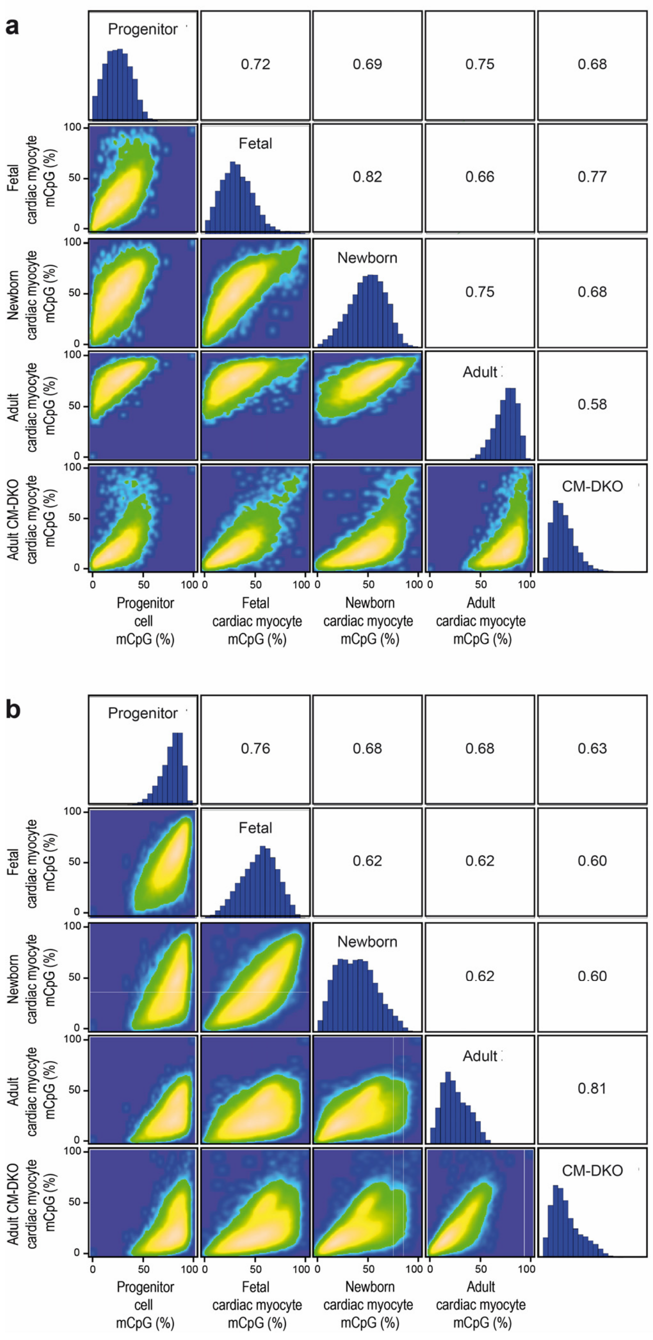

Supplementary Figure 7 │ Histograms of mCpG, scatter plots and Pearson correlation coefficients of developmental DMRs.

a, b, Differentially methylated regions in adult cardiac myocytes as compared to cardiac progenitor cells (**a,** hypermethylated and **b,** hypomethylated regions)**.** Pairwise comparisons of CpG methylation values are displayed as scatter plots and histograms. Values indicate Pearson correlation coefficients. Data was obtained from cardiac progenitor cells and fetal (E14), newborn (P1) and adult cardiac myocytes as well as from adult cardiac myocytes with a cardiac myocytes-specific ablation of DNMT3A and B (CM-DKO, *Dnmt3a*$^{-/-}$/*Dnmt3b*$^{-/-}$). Data shown are merged from n=2-3 replicates (see Supplementary Table 1 and 2).

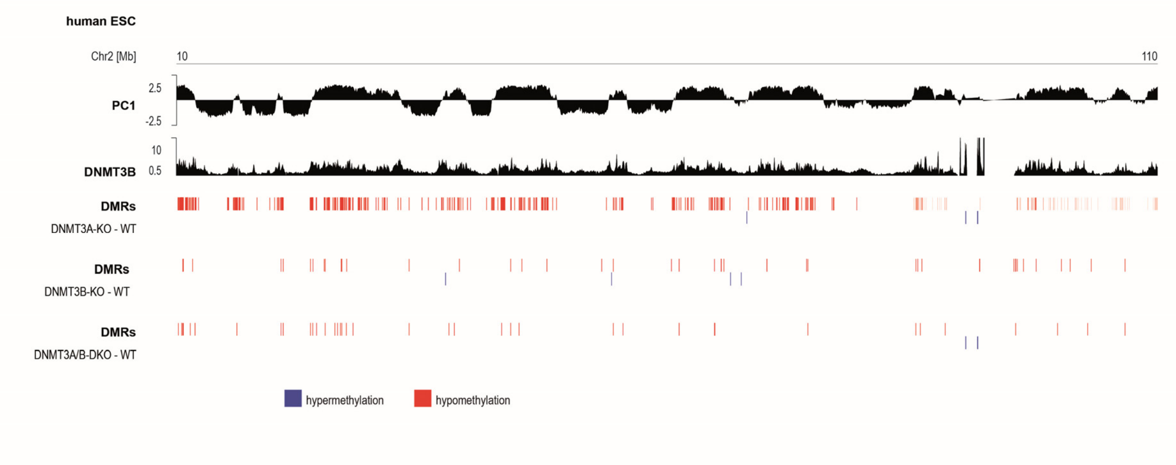

Supplementary Figure 8 │ Differential CpG methylation in huma ESC with ablation of DNMT3 enzymes

Original traces of PC1 values corresponding to A/B compartments (A, PC1 >0; B PC1 < 0), enrichment of DNMT3B and differential CpG methylation (Δ >40%) in human ES cells with ablation of single or both DNMT3 enzymes. Data shown are merged data from n=1-2 replicates (see Supplementary Table 1 and 2).

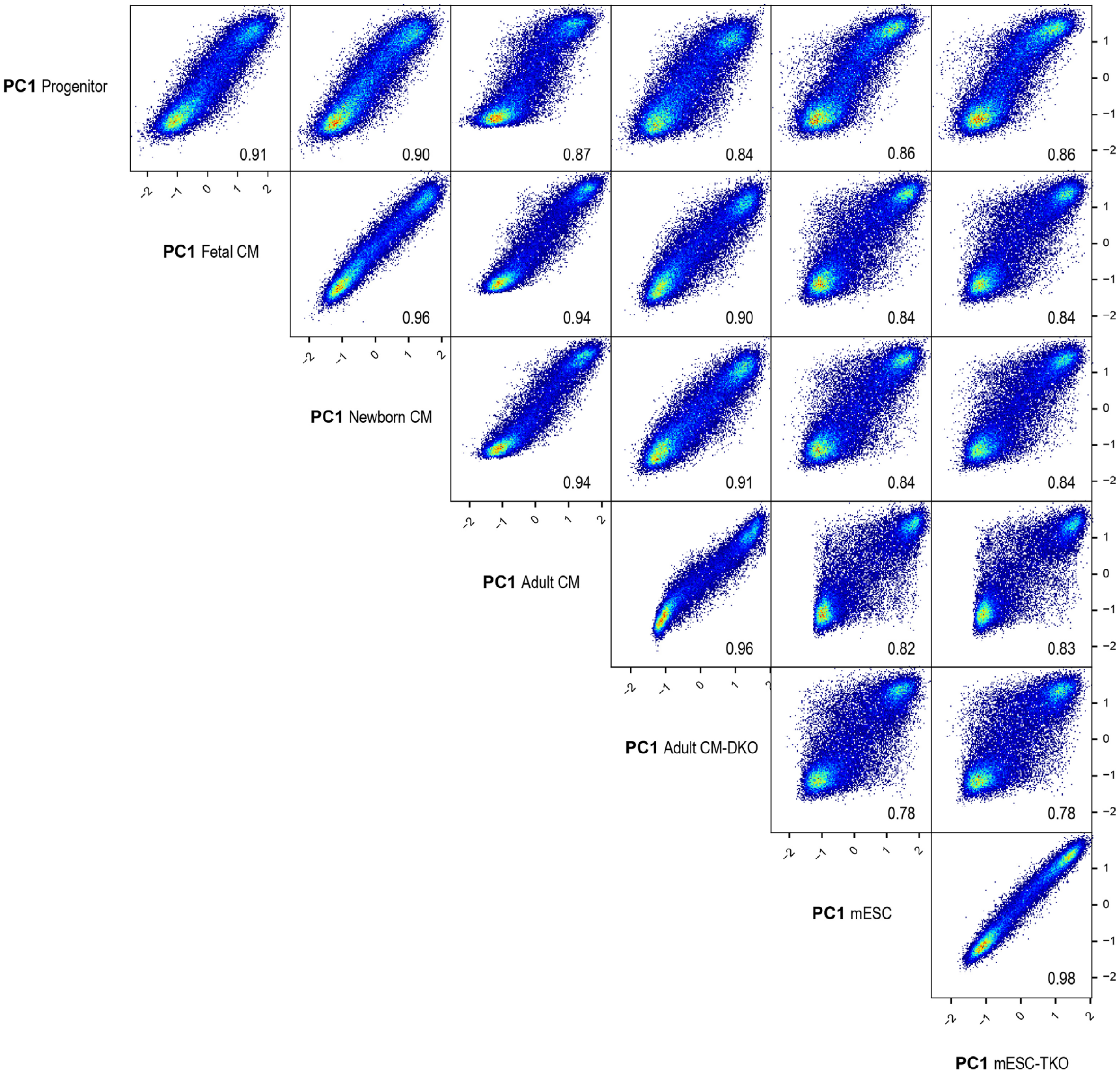

Supplementary Figure 9 │ Correlation of PC1 values

Shown are scatter plots of PC1 values measured in different developmental stages of cardiac myocytes (fetal, E14; newborn, P1; adult) and ESC as well as after ablation of DNMT3A/B in cardiac myocytes (CM-DKO, *Dnmt3a*$^{-/-}$/*Dnmt3b*$^{-/-}$) and DNMT1 and DNMT3A/B in ESC (ESC-TKO, *Dnmt1*$^{-/-}$/*Dnmt3a*$^{-/-}$/*Dnmt3b*$^{-/-}$). Numbers represent Pearson correlation coefficients. Low correlation coefficients were observed between undifferentiated (ES and progenitor cells) and different stages of differentiated CM. Data shown are merged data from n=2-3 replicates (see Supplementary Table 1 and 2).

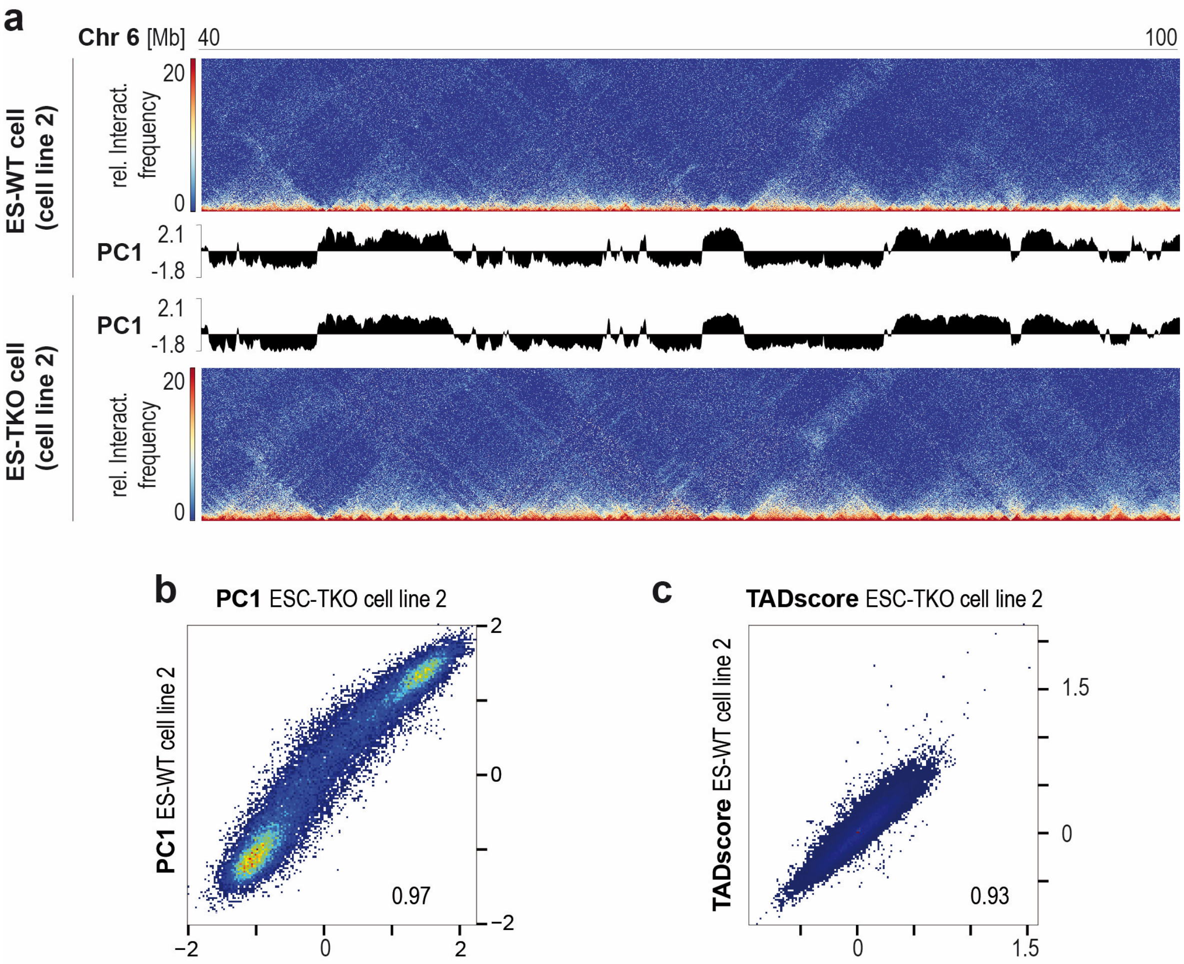

Supplementary Figure 10 │ Analysis of chromatin organization in ES-TKO cell line and corresponding wild type cells generated by Tsumura et al. 2006

a, Chromatin interaction maps of mouse embryonic stem cells (ES cell line 2 , upper panel) and ES cells with a complete loss of DNA methylation (ES-TKO cell line 2, *Dnmt1*$^{-/-}$*/Dnmt3a*$^{-/-}$*/Dnmt3b*$^{-/-}$) are indistinguishable. **b,c,** Genome wide correlation show that ablation of DNMT-isoenzymes has no effect of A-/B-pattern (**b,** scatter plot and Pearson correlation of PC1-values) and insulation of topologically associated domains (**c,** scatter plot and Pearson correlation of TADscore). Data shown represent a replicate of Fig. 4 using independent ES and ES-TKO cell lines (see Supplementary Table 1 and 2).

Supplementary Table 1

Sequencing data generated for this study

experiment	organism	cell	replicate (ID)	aligned data	unit
in situ Hi-C	mouse, C57BL/6J	adult cardiac myocyte	1	1.05E+08	used pairs
in situ Hi-C	mouse, C57BL/6J	adult cardiac myocyte	2	1.15E+08	used pairs
in situ Hi-C	mouse, C57BL/6J	adult cardiac myocyte	3	2.96E+07	used pairs
in situ Hi-C	mouse, C57BL/6J	P1 cardiac myocyte	1	1.23E+07	used pairs
in situ Hi-C	mouse, C57BL/6J	P1 cardiac myocyte	2	1.32E+07	used pairs
in situ Hi-C	mouse, C57BL/6J	E14.5 cardiac myocyte	1	1.34E+07	used pairs
in situ Hi-C	mouse, C57BL/6J	E14.5 cardiac myocyte	2	1.04E+07	used pairs
in situ Hi-C	DNMT3a/b$^{-/-}$	adult cardiac myocyte	1	2.99E+07	used pairs
in situ Hi-C	DNMT3a/b$^{-/-}$	adult cardiac myocyte	2	3.59E+07	used pairs
in situ Hi-C	wildtype (cell line 1)	ES-cell	1	4.54E+07	used pairs
in situ Hi-C	wildtype (cell line 1)	ES-cell	2	4.22E+07	used pairs
in situ Hi-C	wildtype (cell line 2)	ES-cell	3	7.58E+07	used pairs
in situ Hi-C	DNMT1/3a/3b$^{-/-}$ (cell line 1)	ES-cell	1	3.95E+07	used pairs
in situ Hi-C	DNMT1/3a/3b$^{-/-}$ (cell line 1)	ES-cell	2	4.25E+07	used pairs
in situ Hi-C	DNMT1/3a/3b$^{-/-}$ (cell line 2)	ES-cell	3	5.84E+07	used pairs
in situ Hi-C	Nkx2.5-enhancer-EGFP	cardiac progenitor	2	2.17E+07	used pairs
in situ Hi-C	Nkx2.5-enhancer-EGFP	cardiac progenitor	3	2.24E+07	used pairs
WGBS	mouse, C57BL/6J	E14.5 cardiac myocyte	1	9	mean coverage (combined strands)
WGBS	mouse, C57BL/6J	E14.5 cardiac myocyte	2	10	mean coverage (combined strands)
WGBS	mouse, C57BL/6J	Progenitor cell	1	12	mean coverage (combined strands)
WGBS	mouse, C57BL/6J	Progenitor cell	2	9	mean coverage (combined strands)
WGBS	DNMT3a/3b$^{-/-}$	adult cardiac myocyte	1	11	mean coverage (combined strands)
WGBS	DNMT3a/3b$^{-/-}$	adult cardiac myocyte	2	9	mean coverage (combined strands)
RNA-seq	mouse, C57BL/6J	adult cardiac myocyte	1	2.86E+07	uniquely mapped reads
RNA-seq	mouse, C57BL/6J	adult cardiac myocyte	2	2.82E+07	uniquely mapped reads
RNA-seq	mouse, C57BL/6J	adult cardiac myocyte	3	2.82E+07	uniquely mapped reads
RNA-seq	mouse, C57BL/6J	P1 cardiac myocyte	1	2.99E+07	uniquely mapped reads
RNA-seq	mouse, C57BL/6J	P1 cardiac myocyte	2	3.63E+07	uniquely mapped reads
RNA-seq	mouse, C57BL/6J	P1 cardiac myocyte	3	3.43E+07	uniquely mapped reads
RNA-seq	Nkx2.5-enhancer-EGFP	cardiac progenitor	1	7.21E+06	uniquely mapped reads
RNA-seq	Nkx2.5-enhancer-EGFP	cardiac progenitor	2	8.63E+06	uniquely mapped reads
RNA-seq	Nkx2.5-enhancer-EGFP	cardiac progenitor	3	7.21E+06	uniquely mapped reads
RNA-seq	Nkx2.5-enhancer-EGFP	cardiac progenitor	4	7.60E+06	uniquely mapped reads
RNA-seq	DNMT3a/b$^{-/-}$	adult cardiac myocyte	1	2.66E+07	uniquely mapped reads
RNA-seq	DNMT3a/b$^{-/-}$	adult cardiac myocyte	2	2.62E+07	uniquely mapped reads
RNA-seq	DNMT3a/b$^{-/-}$	adult cardiac myocyte	3	1.93E+07	uniquely mapped reads
RNA-seq	wildtype mouse,	ES-cell	1	4.73E+07	uniquely mapped reads
ChIP-seq (CTCF)	C57BL/6J mouse,	adult cardiac myocyte	1	3.70E+07	uniquely mapped reads
ChIP-seq (Cohesin)	C57BL/6J mouse,	adult cardiac myocyte	1	4.04E+07	uniquely mapped reads
ChIP-seq (H3K9me1)	C57BL/6J mouse,	adult cardiac myocyte	1	2.83E+07	uniquely mapped reads
ChIP-seq (H3K9me2)	C57BL/6J mouse,	adult cardiac myocyte	1	3.21E+07	uniquely mapped reads
ChIP-seq (H3K9me3)	C57BL/6J	adult cardiac myocyte	1	3.41E+07	uniquely mapped reads

Published sequencing data from our group

experiment	organism	cell	replicates (n)	accession ID	citation
WGBS	mouse, C57BL/6J	adult cardiac myocyte	3	SRP033288	doi: 10.1038/ncomms6288
WGBS	mouse, C57BL/6J	P1 cardiac myocyte	3	SRP033288	doi: 10.1038/ncomms6288
ChIP-seq (H3K27ac)	mouse, C57BL/6J	adult cardiac myocyte	2	SRX385238, SRX385243	doi: 10.1038/ncomms6288
ChIP-seq (H3K27me3)	mouse, C57BL/6J	adult cardiac myocyte	2	SRX385241, SRX385237	doi: 10.1038/ncomms6288
ChIP-seq (H3K4me1)	mouse, C57BL/6J	adult cardiac myocyte	2	SRX385242, SRX385236	doi: 10.1038/ncomms6288
ChIP-seq (H3K4me3)	mouse, C57BL/6J	adult cardiac myocyte	2	SRX385235, SRX385240	doi: 10.1038/ncomms6288
ChIP-seq (H3K36me3)	mouse, C57BL/6J	adult cardiac myocyte	1	SRP033385	doi: 10.1161/CIRCRESAHA.115.306337
5hmC-seq	mouse, C57BL/6J	adult cardiac myocyte	2	ERS1434318, ERS1434317	doi: 10.1371/journal.pone.0166575

Supplementary Table 2

External data analyzed in this study

experiment	accession number	organism	cell	lab	reference
WGBS	SRX080191, SRX080192	mouse, 129-C57Bl/6	ES	Schübeler	doi: 10.1038/nature10716
ChIP-seq (CTCF)	ENCFF001LIY, ENCFF001LIC	mouse, C57BL/6	ES-Bruce4	Ren	doi: 10.1038/nature13992
ChIP-seq (H3K27ac)	ENCFF001KDQ, ENCFF001K	mouse, C57BL/6	ES-Bruce4	Ren	doi: 10.1038/nature13992
ChIP-seq (H3K27me3)	ENCFF001KED, ENCFF001KI	mouse, C57BL/6	ES-Bruce4	Ren	doi: 10.1038/nature13992
ChIP-seq (H3K36me3)	ENCFF001KEE, ENCFF001KI	mouse, C57BL/6	ES-Bruce4	Ren	doi: 10.1038/nature13992
ChIP-seq (H3K4me1)	ENCFF001KEF, ENCFF001KI	mouse, C57BL/6	ES-Bruce4	Ren	doi: 10.1038/nature13992
ChIP-seq (H3K4me3)	ENCFF001KER, ENCFF001KI	mouse, C57BL/6	ES-Bruce4	Ren	doi: 10.1038/nature13992
ChIP-seq (H3K9me3)	ENCFF001KDP, ENCFF001KI	mouse, C57BL/6	ES-Bruce4	Ren	doi: 10.1038/nature13992
Hi-C	SRX378271	human	H1 ES	Ren	doi: 10.1038/nature14222
ChIP-seq (DNMT3B)	SRX156112	human	undiff. NCCIT	Robertson	doi:10.1016/j.celrep.2012.10.017
5hmC-seq	SRX057744	mouse	E14 ES	Wu	doi:10.1016/j.molcel.2011.04.005
ChIP-seq (Cohesin (SMC1/	SRX022688, SRX022689	mouse, C57BL/6-129	V6.5, ES	Young	doi:10.1038/nature09380
ChIP-seq (TET1)	SRX039347	mouse, E14Tg2A	ES	Zhang	doi:10.1038/nature09934
ChIP-seq (CTCF-TKO)	SRX080170, SRX080171	mouse, 129-C57Bl/6	ES	Schübeler	doi:10.1038/nature10716
ChIP-seq (CTCF-WT)	SRX080167, SRX080168, SR	mouse, 129-C57Bl/6	ES	Schübeler	doi:10.1038/nature10716
Input	SRX080178, SRX080179	mouse, 129-C57Bl/6	ES	Schübeler	doi:10.1038/nature10716
ChIP-seq (DNMT3A)	SRR1274746, SRR1274747	mouse, 129-C57Bl/6	ES-HA36CB1	Schübeler	doi:10.1038/nature14176
ChIP-seq (DNMT3B)	SRR1274748, SRR1274749	mouse, 129-C57Bl/6	ES-HA36CB1	Schübeler	doi:10.1038/nature14176
WGBS (DNMT3B-KO)	SRX759480, SRX759483	human	HUESC64	Meissner	doi:10.1038/ng.3258
WGBS(DNMT3A/3B-DKO)	SRX759481, SRX759484	human	HUESC64	Meissner	doi:10.1038/ng.3258
WGBS(DNMT3A-KO)	SRX759479, SRX759482	human	HUESC64	Meissner	doi:10.1038/ng.3258
